# Radio frequency plasma assisted surface modification of Fe_3_O_4_ nanoparticles using polyaniline/polypyrrole for bioimaging and magnetic hyperthermia applications

**DOI:** 10.1007/s10856-021-06563-1

**Published:** 2021-08-25

**Authors:** Beena Mol, Ansar Ereath Beeran, Prasad S. Jayaram, Prabha Prakash, Ramapurath S. Jayasree, Senoy Thomas, Baby Chakrapani, M. R. Anantharaman, M. Junaid Bushiri

**Affiliations:** 1 Department of Physics, Cochin University of Science and Technology, Cochin, Kerala 682022 India; 2 Bioceramics Laboratory, Sree Chitra Tirunal Institute for Medical Sciences & Technology, Poojappura, Kerala 695012 India; 3 Department of Chemistry, M.E.S Asmabi College, P. Vemballur, Kodungallur, Thrissur, Kerala 680671 India; 4 Division of Biophotonics and Imaging, Biomedical Technology Wing, Sree Chitra Tirunal Institute for Medical Sciences & Technology, Poojappura, Thiruvananthapuram, Kerala 695012 India; 5 Centre for Neuroscience, Cochin University of Science and Technology, Cochin, Kerala 682022 India; 6 Department of Biotechnology, Cochin University of Science and Technology, Cochin, Kerala 682022 India

## Abstract

Surface modification of superparamagnetic Fe_3_O_4_ nanoparticles using polymers (polyaniline/polypyrrole) was done by radio frequency (r.f.) plasma polymerization technique and characterized by XRD, TEM, TG/DTA and VSM. Surface-passivated Fe_3_O_4_ nanoparticles with polymers were having spherical/rod-shaped structures with superparamagnetic properties. Broad visible photoluminescence emission bands were observed at 445 and 580 nm for polyaniline-coated Fe_3_O_4_ and at 488 nm for polypyrrole-coated Fe_3_O_4_. These samples exhibit good fluorescence emissions with L929 cellular assay and were non-toxic. Magnetic hyperthermia response of Fe_3_O_4_ and polymer (polyaniline/polypyrrole)-coated Fe_3_O_4_ was evaluated and all the samples exhibit hyperthermia activity in the range of 42–45 °C. Specific loss power (SLP) values of polyaniline and polypyrrole-coated Fe_3_O_4_ nanoparticles (5 and 10 mg/ml) exhibit a controlled heat generation with an increase in the magnetic field.

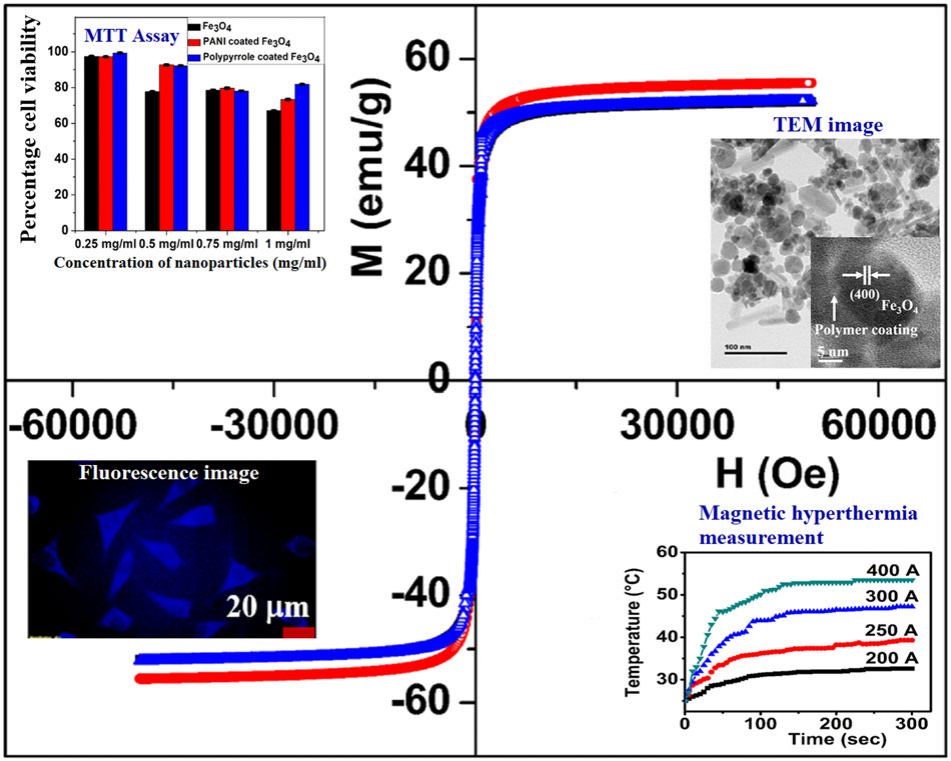

## Introduction

Magnetic, optical, semiconducting and biocompatible properties of magnetite (Fe_3_O_4_) are tunable, which make it as a promising candidate for magnetic resonance imaging, targeted drug delivery and cancer therapy [1–7]. Magnetite nanoparticles can be functionalised in a relatively easy way and this makes it an ideal candidate for various biological applications [1]. Controlling the particle size and subsequent surface modification to achieve a core-shell nanostructure makes it a synergetic multifunctional material for imaging and as a therapeutic agent in cancer therapy [8–11]. However, Fe_3_O_4_ nanoparticles easily get agglomerate due to their inter-nanoparticle magnetic dipolar interactions and low surface potential and will adversely affect their useful magnetic properties. Agglomeration of Fe_3_O_4_ nanoparticles can be prevented to a certain extent by coating a thin layer of nonmagnetic materials like graphite, polyethylene, polyvinyl alcohol, polyaniline and silica [2, 12]. Previous studies reported the use of cross-linked polymers as a surface coating for Fe_3_O_4_ nanoparticles, which resulted in the modification of its magnetic, optical and physical properties [13, 14].

Superparamagnetic Fe_3_O_4_ nanoparticles release thermal energy when subjected to an alternating magnetic field which is due to the Brownian and Neel relaxation [5, 15]. This property of superparamagnetic iron oxide nanoparticles (SPIONs) can be used for hyperthermia generation and is effective for the treatment of tumours [7]. It is reported that cancerous cells are more sensitive to temperature in the range of 42–45 °C than normal tissues [6]. However, studies show that an uncontrolled increase in temperature up to 60 °C leads to irreversible cell injury and damage to the healthy tissue [16, 17]. Therefore, a well-controlled heat generating system is required for initiating desired death of malignant cells by hyperthermia therapy. Heating efficiency of the superparamagnetic nanoparticles suspended in a medium depends on external parameters like frequency, strength of external alternating magnetic field and internal intrinsic properties (core size, shape, shell thickness, colloidal stability) of the material [18, 19]. Fine tuning of these parameters will help to develop new combination of materials with improved heating efficiency suitable for magnetic hyperthermia therapy. Among the superparamagnetic materials, Fe_3_O_4_ and γ-Fe_2_O_3_ has the advantage of providing better heating rates with minimum dosage and can also retain smooth circulation in the bloodstream [18, 19].

The hyperthermia response of the Fe^0^/PANI/polycaprolactone nanofibres synthesized by electrospinning is reported previously [20]. Eddy current loss from PANI and Neel’s relaxation from magnetic Fe^0^ nanoparticles together contribute to the power dissipation in composite fibres and provide better heating efficiency in this system [20]. Beeran et al. observed an enhancement in hyperthermia activity in *HeLa* cell population using ferrofluid-based superparamagnetic iron oxide substituted with Mn^2+^ and stabilized using trisodium citrate surface coating [21]. Fe_3_O_4_ nanoparticles showed a decrease in the magnetic moment and saturation magnetization on coating with a thermosensitive polymer, poly(2-(dimethylamino)ethyl methacrylate) (PDMAEMA) [22]. However, an enhancement in the heating efficiency of PDMAEMA coated nanoparticles is observed, compared to bare Fe_3_O_4_ nanoparticles [22]. Nemati et al. synthesized iron oxide nano-octopodes by thermal decomposition and studied its hyperthermia response [23]. A large improvement in heating efficiency (up to 70%) was obtained by changing the size and shape of the nanoparticles [23]. Studies indicate that higher heating rates for magnetic hyperthermia can be achieved by increasing the Brownian relaxation (since *V*_H_ ≫ *V*_M_) [18, 19]. SPIONs coated with polymers can heat better due to increase in Brownian relaxation even at higher concentration and help to generate temperature elevation in a controlled manner.

Apart from the magnetic and hyperthermia properties, Fe_3_O_4_ is also a semiconducting material in which intervalence and intersublattice charge transfer occur on optical excitation, which contributes to its light emission properties [24]. Similar to other semiconducting materials, presence of defects, oxygen vacancies or excitons are responsible for the optical properties of these Fe_3_O_4_ [25]. One of the major challenges in the synthesis of magnetic-fluorescent nanomaterials is the quenching of photoluminescence (PL) emission by the magnetic core, contributed to the electron transfer between Fe^2+^ and Fe^3+^ ionic states in the tetrahedral and octahedral sites with the ligands [24–27]. This type of PL quenching can be controlled by coating the ferrite using polymers/silica layer [26, 27]. Passivation of magnetite nanoparticles using conjugated polymers can also serve as a good photothermal agent for cancer therapy for both in vivo and in vitro applications [9, 10]. Moreover, the conjugated polymer backbone can act as an array of light-harvesting units, having larger optical cross-sections compared to other organic dyes [8]. The modifications of magnetic, semiconducting and optical properties by encapsulation of magnetite with polymers result in efficient fluorescent probes having both imaging and targeting capabilities.

Various methods like chemical co-precipitation, sol-gel method, radio frequency (r.f.) plasma polymerization etc. can be adopted to provide a thin layer of polymer coating to superparamagnetic Fe_3_O_4_ [1, 13]. Among these methods, r.f. plasma polymerization is a single-step and cost-effective technique. Plasma treated surface modifications promote adhesion between polymer and Fe_3_O_4_ nanoparticles, and contributes hydrophilicity to the composite [28]. The plasma process also ensures a sterile surface for the as synthesized material which makes the material more suitable for biological applications due to its biocompatibility [28, 29]. The thickness of the coating (~few nm) over the material can be controlled by monitoring the monomer flow rates, pressure, current and time of deposition. Sethulakshmi et al. reported an enhancement in the saturation magnetization of polyaniline (PANI) coated ferromagnetic magnetite and γ- Fe_2_O_3_ spherical nanoparticles having a size of 20–30 nm prepared by r.f. plasma polymerization [13].

Various reports indicate that thin polymer coating over bare Fe_3_O_4_ nanoparticles adds multi-functionality, reduces agglomeration and provides better dispersibility in the aqueous media [13, 22]. In this context, the present work describes the synthesis and surface modification of superparamagnetic Fe_3_O_4_ nanoparticles with polyaniline and polypyrrole by r.f. plasma polymerization process. Magnetic and PL/fluorescence emission properties, magnetic hyperthermia response and cytotoxicity of the as synthesized samples are also investigated.

## Materials and methods

### Preparation of iron oxide nanoparticles by co-precipitation method

Superparamagnetic Fe_3_O_4_ nanoparticles were prepared by controlled co-precipitation method [30]. For this, 0.2 M FeCl_3_ and 0.1 M FeSO_4_.7H_2_O (Merk) each in 120 ml prepared in distilled water were taken and stirred for 30 min. The pH was adjusted to 10 by adding drop-wise aqueous ammonia into the above solution at room temperature. The resulting solution was heated to a temperature of 80 °C for about 90 min. This sample was then washed with distilled water several times for the removal of water soluble byproducts and kept it for gravity settling of nanoparticles. It was again washed with distilled water and centrifuged (3500 rpm). The residue obtained was black in colour and kept for drying at 80 °C for 3 h in a hot air oven.

### Surface modification of iron oxide nanoparticles using r.f. plasma polymerization method

Fe_3_O_4_ nanoparticles were coated with polymers by taking double-distilled monomers (aniline/pyrrole) with r.f. plasma polymerization process by using a home-made r.f. plasma polymerization setup [13, 14]. It consists of a borosilicate glass tube of dimension (length 50 cm, diameter 5 cm) with provisions for evacuation as well as an inlet for monomer. The r.f. plasma polymerization chamber was evacuated (0.028 millibar) using a rotary pump after placing Fe_3_O_4_ powder inside the chamber in between the copper electrodes separated between a distance of 5 cm. In between the electrodes, a glow discharge of plasma was produced by providing an r.f. frequency of 7–13 MHz. The current for generating plasma was maintained at 74 mA and pressure inside the chamber was 0.032 millibar after monomer injection. Iron oxide powder placed inside the chamber exactly in between the electrodes was continuously stirred using a magnetic stirrer placed below the chamber, so that magnetic grains belonging to the experimental sample are renewed and exposed to plasma during polymerization. Monomer sprayed in the region of plasma undergoes polymerization process and deposited on the surface of the iron oxide nanoparticles. The deposition process was carried out for about 7 min inside the chamber. After that, the sample was taken out from the reactor and washed with distilled water several times and kept for drying at 80 °C. The sample thus obtained was powdered using a mortar and pestle, and used for further studies. Both PANI-coated Fe_3_O_4_ and polypyrrole-coated Fe_3_O_4_ samples were prepared with above-mentioned r.f. plasma polymerization setup.

### Characterizations

X-ray diffraction of the samples was carried out using Bruker D8 Advance Twin-Twin equipment using Cu-K_α_ (1.5404 Å) radiation. Powdered samples were dispersed and pressed on the glass plate before taking XRD of the sample. Transmission electron microscopy images of the samples were taken using JEOL JEM 2100 HRTEM operating at 200 kV. Powdered samples were dispersed in ethanol and sonicated and were taken on a carbon grid before TEM measurements. Thermogravimetric analysis (TGA) was performed with a Perkin Elmer, Diamond TG/DTA in a nitrogen atmosphere in the temperature range 15–900 °C at 20 °C/min. Vibrating Sample Magnetometer measurements of the powder samples were performed using Quantum Design Dynacool PPMS in the field range ±50 KOe at 300 K. PL studies of the samples were investigated using an inVia Reflex Raman spectrometer attached with a laser of excitation wavelength 405 nm (Renishaw, UK, Model No. M-9836-3991-01-A).

### Biocompatibility studies using MTT assay

For cell studies, samples were sterilized initially by keeping under UV light (30 W UV lamp of intensity 337.5 lumen) for 4 h. The U87 cells (Glioma cells) were seeded at a density of 10^4^ cells ml^−1^ in a flat bottomed 96-well polystyrene-coated plate and was incubated at 37 °C for 24 h in a 5% CO_2_ incubator. Different concentrations (0.25, 0.5, 0.75 and 1 mg/ml) of Fe_3_O_4_, PANI- and polypyrrole-coated Fe_3_O_4_ in the medium was added to the plates in hexaplets. This system was kept for 24 hrs incubation, 20 μl (5 mg/ml) MTT (3-(4, 5-dimethylthiazol-2-yl)-2, 5-diphenyltetrazolium bromide) solution was added to each well which is incubated for another 3 hrs. Formazan crystals were solubilised in dimethyl sulphoxide and absorbance was monitored at 595 nm in a microplate reader (BIO-RAD iMark^TM^Microplate Reader). Wells with complete media, nanoparticles and MTT reagent without cells were used as control. The untreated U87 cells and cells treated for 24 hrs with the above-mentioned concentration of nanoparticles were used for the MTT assay for cell viability determination. Percentage cell viability was calculated using the following formula [31]:

1 $${{{\rm{Cell}}}\,{{\rm{viability}}}}\,\left( \% \right) = \frac{{{{\rm{Absorption}}}\,test}}{{{{\rm{Absorption}}}\,{{\rm{control}}}}} \times 100$$

### Uptake studies

L929 cell lines were used for the cellular uptake studies of the nanoparticles, fluorescence images were taken using DAPI excitation. The cells were initially seeded on to culture plates, which were allowed to reach 80% confluency by incubating the plates in CO_2_ incubator at 37 °C. The cells were treated with 0.5 mg/ml concentration of respective samples (Fe_3_O_4_, PANI-coated Fe_3_O_4_ and polypyrrole-coated Fe_3_O_4_) since this concentration gives the best cell viability as understood from MTT assay. Wells were treated with the material for different time intervals ranging from 1, 2, 4 h. The aliquots were made in distilled water, since all the samples are dispersible in water. Bright field and fluorescent images (×40 magnification) of the samples were taken after the incubation of the materials with the cells using Olympus IX73 optical imaging system. A control experimental system was maintained for comparison with the treated materials.

### Hyperthermia studies

The suitability of materials for hyperthermia applications was studied using Ambrell EASY HEAT induction system. The experimental setup consists of a solenoid coil (6 turns) with a diameter of 4 cm and length 2.6 cm in which ac (alternating current) frequency was set to 280 kHz. The heating efficiency of the sample at different concentrations (1 and 3 mg) and current (alternating magnetic field) were evaluated for 5 min (300 s) [21]. Calculated magnetic field (H) value for current strength 200, 250, 300, 350 and 400 A were 14.4, 19.33, 24.17, 28.99 and 33.83 mT respectively. A non-contact mode IR thermometer (FLUKE 572) with an accuracy of ±1 °C, was used to monitor the temperature of the system as a function of time. For SLP calculation, Fe_3_O_4_, polyaniline (PANI) and polypyrrole-coated Fe_3_O_4_ nanoparticles (5 and 10 mg) concentration were suspended in distilled water in an eppendorf tube (1.5 ml) with a final volume of 250 μl. An alternating field (14.4, 19.33, 24.17, 28.99 and 33.83 mT) was applied with a frequency of 280 kHz to the sample for 3 min.

## Results and discussion

**Fig. 1:**
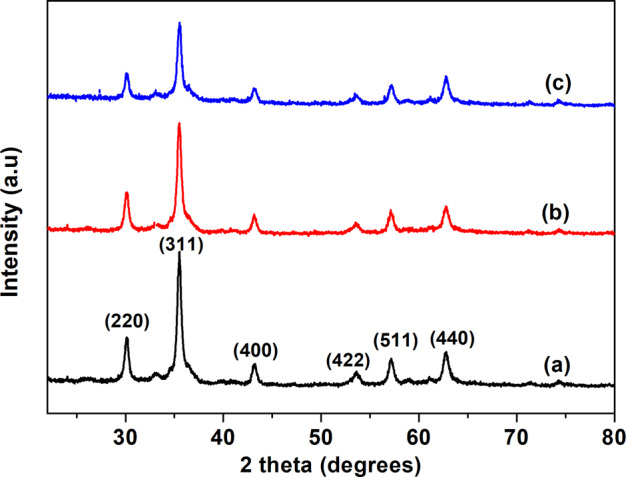
XRD pattern of **a** Fe_3_O_4_, **b** PANI-coated Fe_3_O_4_, and **c** polypyrrole-coated Fe_3_O_4_ nanoparticles

XRD pattern of Fe_3_O_4_ (Fig. 1) shows peaks at 2Ѳ = 30°, 35°, 43°, 53°, 57° and 62° corresponding to (220), (311), (400), (422), (511) and (440) planes of cubic spinel structured Fe_3_O_4_ (ICSD No. 01-071-6336). Polyaniline and polypyrrole-coated Fe_3_O_4_ shows diffraction peaks at the same position as that of Fe_3_O_4_ with slight changes in its intensity. This observation indicates that surface modification of iron oxide nanoparticles by r.f. plasma polymerization is not making appreciable changes in the crystalline structure of Fe_3_O_4_. The presence of diffraction peaks in all the samples indicates the crystalline nature of the sample under investigation and the lattice constant (8.37 Å) of PANI and polypyrrole-coated Fe_3_O_4_ is same as that of Fe_3_O_4_. The intensity ratio of (311) to (220) peak are compared, the intensity of polymer-coated sample is relatively less with that of Fe_3_O_4_, which indicates the reduction in crystallinity of polymer-coated sample.

TGA and differential thermal analysis (DTA) of Fe_3_O_4_ nanoparticles show two stages of thermal decomposition below 153 °C (5%) corresponding to the escape of adsorbed water molecules, and at 251 °C (10%) due to the exothermic phase transition of Fe_3_O_4_ to gamma phase of Fe_2_O_3_ (Supplementary Figs. S1 and S2) [31]. The inert residue obtained from the uncoated Fe_3_O_4_ sample, at 750 °C is calculated as 0.97 mg [Supplementary Fig. S1]. The observed degradation at 66 °C of PANI-coated Fe_3_O_4_ and at 91 °C of polypyrrole-coated Fe_3_O_4_ is due to the escape of adsorbed water molecules similar to that of Fe_3_O_4_. Fe_3_O_4_ to γ‑Fe_2_O_3_ transformation is also observed in both the samples in the range of ~ 237 to 285 °C. DTA curve also shows the transformation peak of Fe_3_O_4_ to γ‑Fe_2_O_3_ at 251 °C (Fe_3_O_4_), 254 °C (PANI-coated Fe_3_O_4_) and 262 °C (polypyrrole-coated Fe_3_O_4_). Later a gradual decrease in weight loss is observed in both PANI and polypyrrole-coated Fe_3_O_4_ samples is attributed to the decomposition of carbonaceous species from the polymer coating. The exothermic peak observed at 406 °C in the DTA curve of PANI-coated Fe_3_O_4_ and 438 °C in polypyrrole-coated Fe_3_O_4_ is related to the escape of polymer related components from this sample [31, 32]. These observations demonstrate that Fe_3_O_4_ phase of iron oxide is stable up to 250 °C. From the TGA curve (Supplementary Fig. S1), the polymer component percentage present in the sample is estimated, which is 4.28% (283–456 °C) in PANI-coated Fe_3_O_4_ and 3.48% (296–519 °C) in polypyrrole-coated Fe_3_O_4_.

**Fig. 2:**
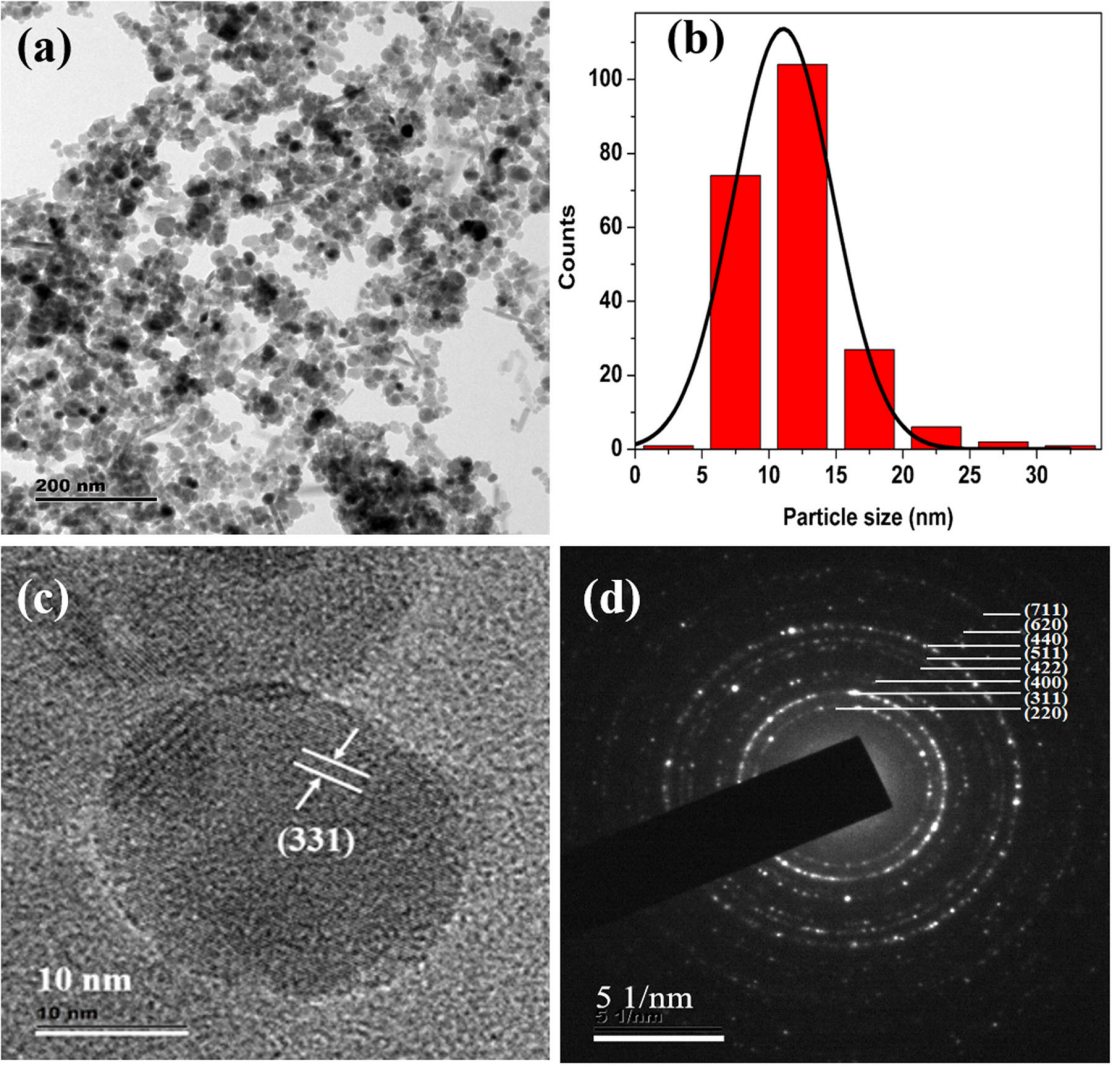
**a** TEM image of Fe_3_O_4_, **b** Histogram of particle sizes obtained from TEM, **c** HRTEM image and **d** SAED pattern of Fe_3_O_4_

TEM images show the presence of spherical particles along with a few rod-shaped iron oxide nanostructures (Fig. 2a), some of these nanostructures are agglomerated. The average particle size of spherical-shaped Fe_3_O_4_ nanoparticles is ~11 nm (Fig. 2b) and that of PANI-coated Fe_3_O_4_ is ~14 nm (Fig. 4b). But spherical structures in the case polypyrrole-coated Fe_3_O_4_, the average particle size is ~18 nm for (Fig. 5b). The rod-shaped structures seen in the TEM images are having length varying between 25–100 nm with an average diameter of ~6 nm (Fe_3_O_4_), 7 nm (PANI-coated Fe_3_O_4_) and 9 nm (polypyrrole-coated Fe_3_O_4_). Spherical particles (~479) are more in number with respect to rod-shaped particles (~95) which are quantified in terms of its shape from the TEM images of PANI-coated Fe_3_O_4_ nanoparticles.

**Fig. 3:**
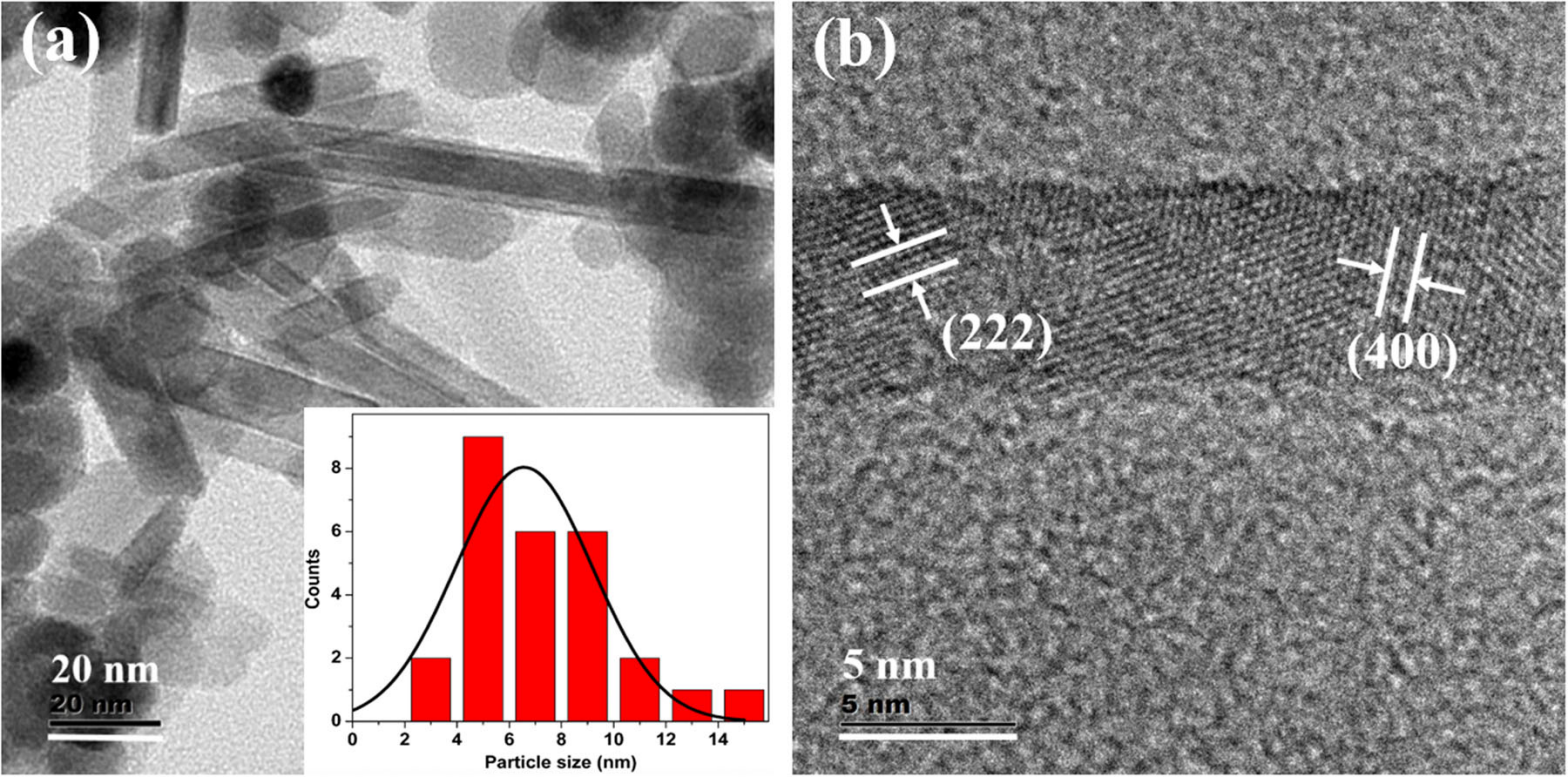
**a** TEM image of iron oxide nanoparticles with spherical and rod-shaped morphology (Fe_3_O_4_). The inset shows the average diameter of nanorods and **b** HRTEM image of nanorod iron oxide

**Fig. 4:**
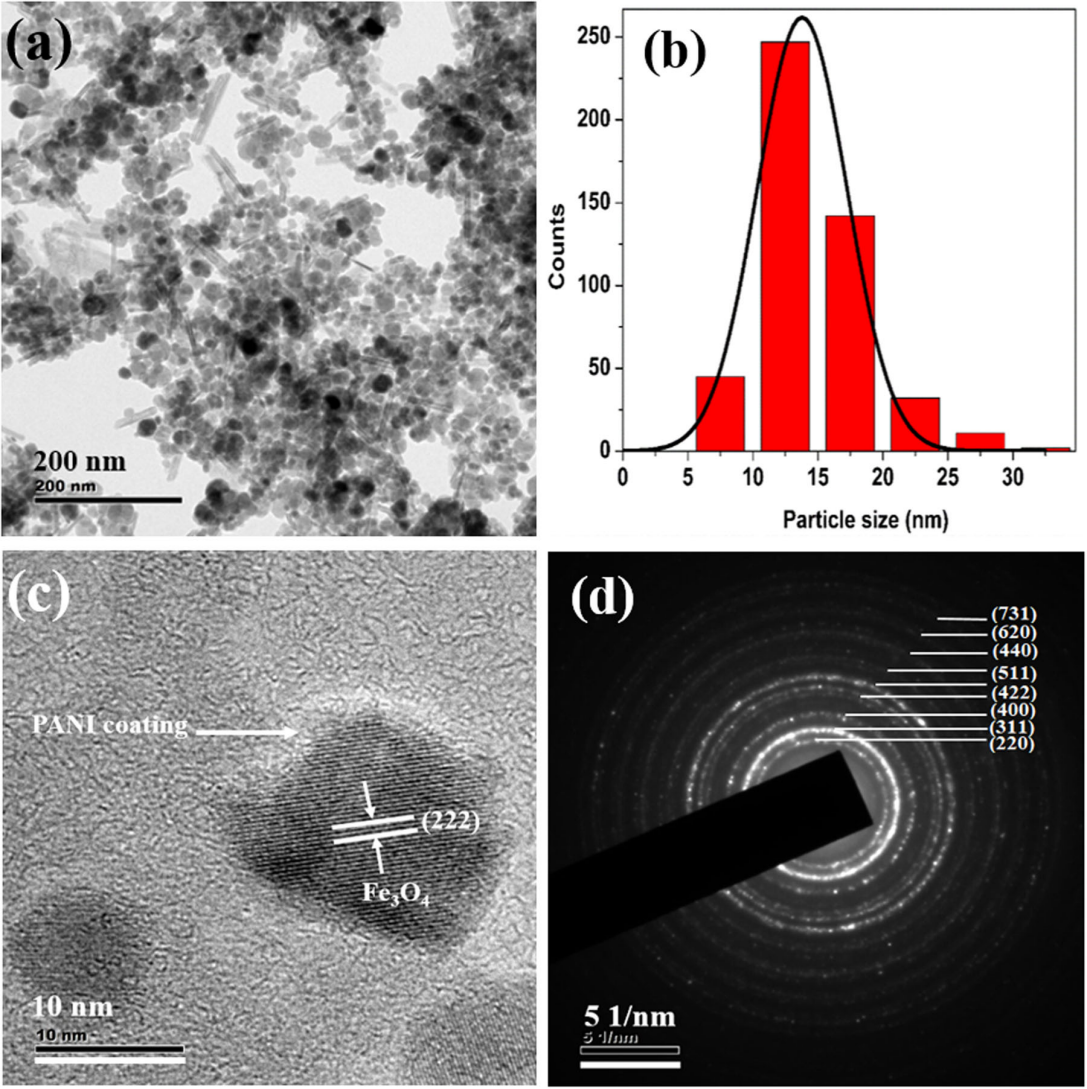
**a** TEM image of PANI-coated Fe_3_O_4_, **b** histogram of particle sizes obtained from TEM, **c** HRTEM image and **d** SAED pattern of PANI-coated Fe_3_O_4_

**Fig. 5:**
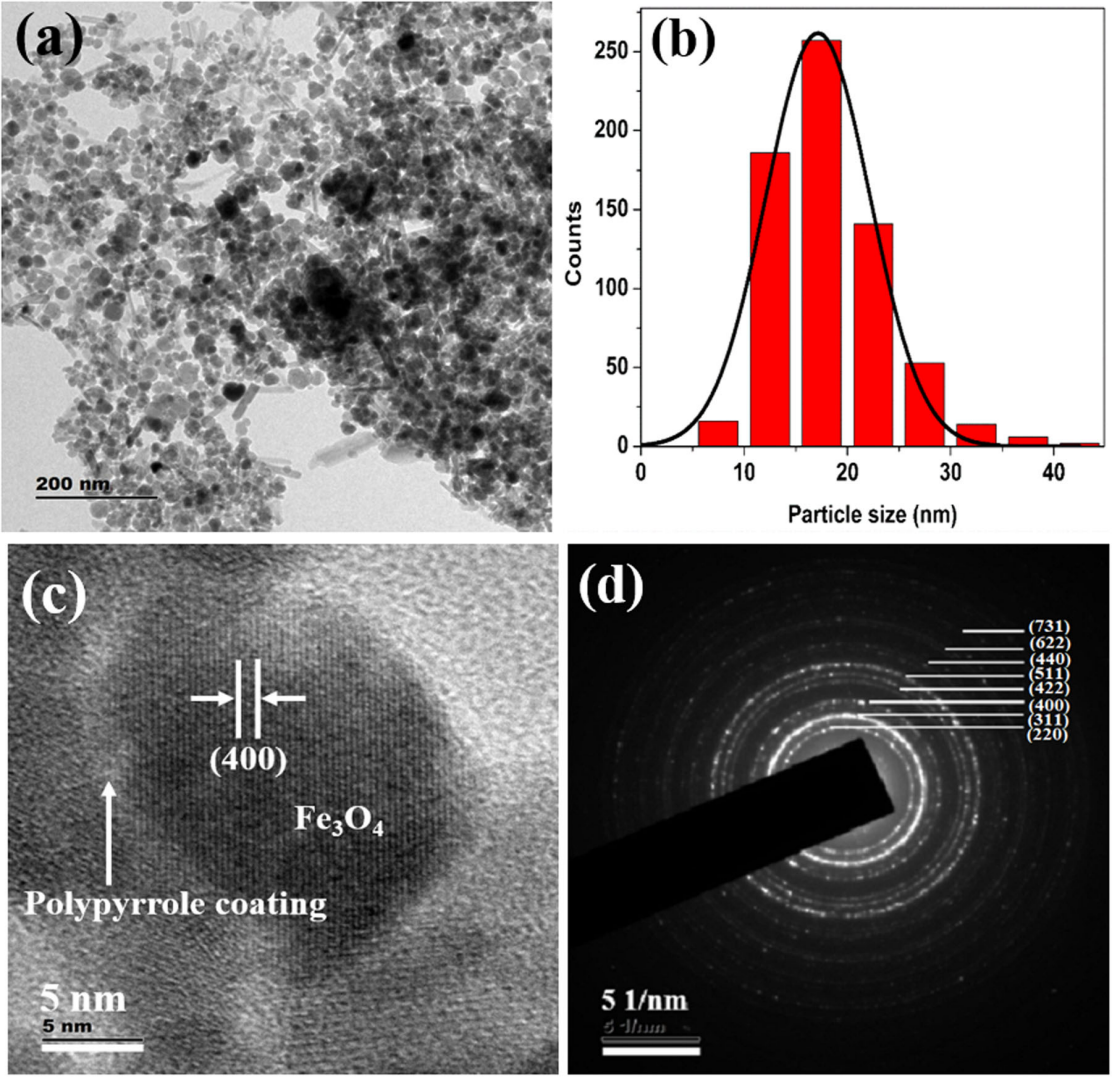
**a** TEM image of polypyrrole-coated Fe_3_O_4_, **b** histogram of particle sizes obtained from TEM, **c** HRTEM image and **d** SAED pattern of polypyrrole-coated Fe_3_O_4_

HRTEM analysis shows that spherical, as well as rod-shaped particles, possess face centred cubic lattice of spinel magnetite. Lattice planes of Fe_3_O_4_ are visible in all the HRTEM images of Fe_3_O_4,_ PANI-coated Fe_3_O_4_ and polypyrrole-coated Fe_3_O_4_ (Figs. 2c, 3b, 4c and 5c)). A thin layer of ~2 nm thickness is seen around the iron oxide nanoparticles in both PANI and polypyrrole-coated iron oxide samples (Figs. 4c and 5c). Sample Fe_3_O_4_ shows (331) planes of Fe_3_O_4_, whereas that of PANI-coated sample shows (222) planes of Fe_3_O_4_. But in the case of polypyrrole-coated sample, (400) planes of Fe_3_O_4_ are more visible. The interplanar spacing (d) obtained from the HRTEM images matches with the lattice planes of Fe_3_O_4_ (ICDD No.85-1436) of spinel magnetite. Lattice planes (222) and (400) of Fe_3_O_4_ are visible for particles with rod-shaped morphology in Fe_3_O_4_ (Fig. 3b).

The selected area electron diffraction (SAED) patterns of all the samples display ring pattern which indicates polycrystalline nature (Figs. 2d, 4d and 5d). Crystalline planes are indexed to fcc lattice of spinel magnetite (Fe_3_O_4_) (ICDD No. 85-1436) are in agreement with XRD results.

**Fig. 6:**
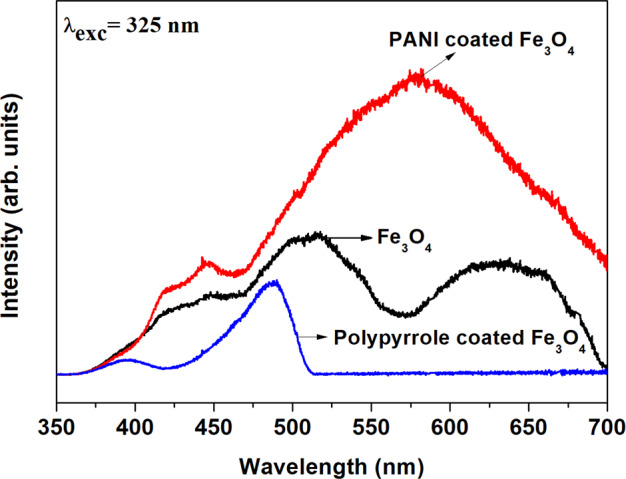
Photoluminescence spectra of Fe_3_O_4_, PANI-coated Fe_3_O_4_ and polypyrrole-coated Fe_3_O_4_ under an excitation wavelength of 325 nm

PL spectra of Fe_3_O_4_, PANI-coated Fe_3_O_4_ and polypyrrole-coated Fe_3_O_4_ at an excitation wavelength of 325 nm is shown in Fig. 6. Fe_3_O_4_ sample shows emission peaks at 513 and 632 nm. PANI-coated Fe_3_O_4_ shows peaks at 445 nm and an intense broad emission peak at 580 nm respectively. Whereas polypyrrole-coated sample exhibits strong peak at 488 nm. Broad PL emission in all the samples indicates the presence of different types of defects in the sample under investigation. Sadat et al. also reported the PL emission in the almost same region in PAA/ Fe_3_O_4_, PS/ Fe_3_O_4_ and Si/PS/ Fe_3_O_4_ nanosystems [25].

PL emission band observed at 513 nm is associated with radiative recombination of electrons from the crystal field site of Fe_3_O_4_ to its octahedral site [25]. The broad peak at 632 nm can be attributed to the recombination of electrons trapped in the octahedral site to O(2p) or from crystal field site to O(2p) on the tetrahedral site [33].

PL emission from the PANI and polypyrrole-coated Fe_3_O_4_ samples is dominated by the polymer shell. Relatively weak emission peak observed at 445 nm (PANI-coated Fe_3_O_4_) and peak at 398 nm (polypyrrole-coated Fe_3_O_4_) arises due to the π−π* transitions occurring in the benzoid units of the polymer chain [34]. The strong PL emission band observed around 580 nm from the PANI-coated Fe_3_O_4_ is attributed to the direct interband transitions occurs in the polymer chain [35]. Another broad emission band at 488 nm of polypyrrole-coated Fe_3_O_4_ contributed to de-excitations from the polaron band [34]. Shift in emission bands observed in the PANI and polypyrrole-coated samples is probably due to the presence of polymer chain aggregation formed during r.f. plasma polymerization process.

**Fig. 7:**
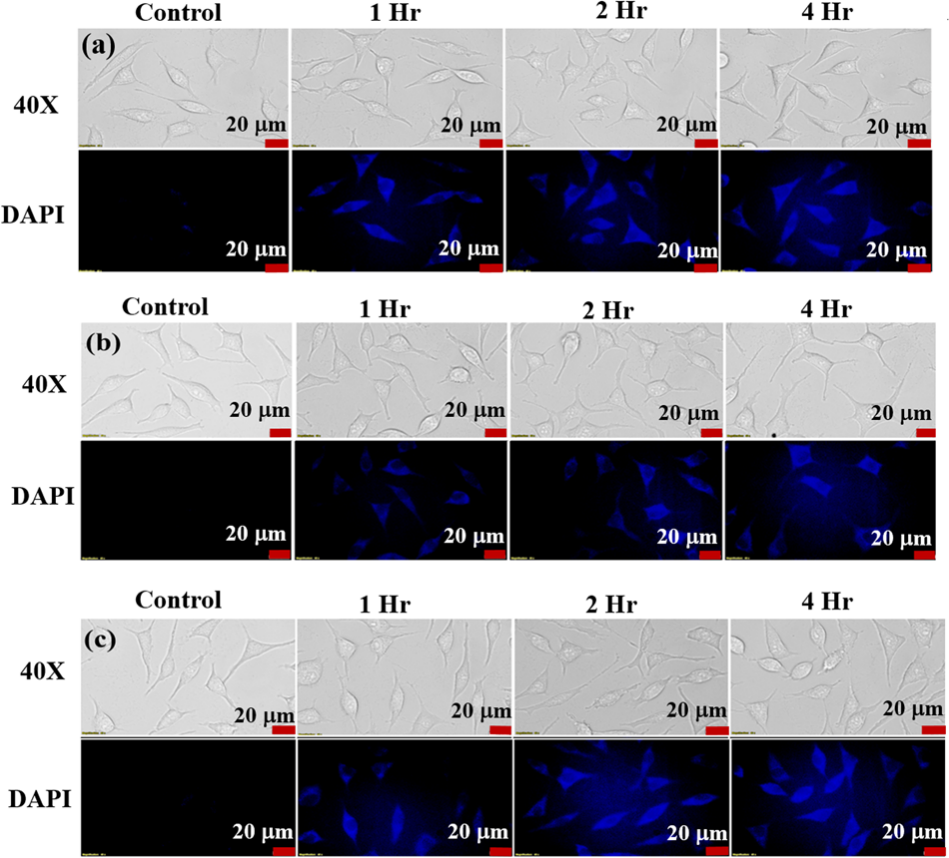
Bright field and fluorescence images of L929 cell lines treated with **a** Fe_3_O_4_, **b** polyaniline-coated Fe_3_O_4_ nanoparticles, and **c** polypyrrole-coated Fe_3_O_4_ nanoparticles (×40 magnification)

Cellular uptake studies of the Fe_3_O_4_, PANI and polypyrrole-coated Fe_3_O_4_ nanoparticles are carried out in normal cell line, L929 (Fig. 7). Fluorescence emissions from the cellular assay at different time intervals are compared, maximum uptake of the samples are observed after 4 h of treating the L929 cell with the samples. One can see from the fluorescence images that there occurs a gradual increase in the fluorescence emission from the cells from the lowest to highest time interval. The bright-field images obtained also show that the cell morphology is not much varied and cells are found to be healthy even after 4 h of incubation. These observations show that Fe_3_O_4_, PANI and polypyrrole-coated Fe_3_O_4_ nanoparticles are non-toxic and its fluorescence properties without the addition of any labelling agents or dyes can be exploited for bioimaging applications.

**Fig. 8:**
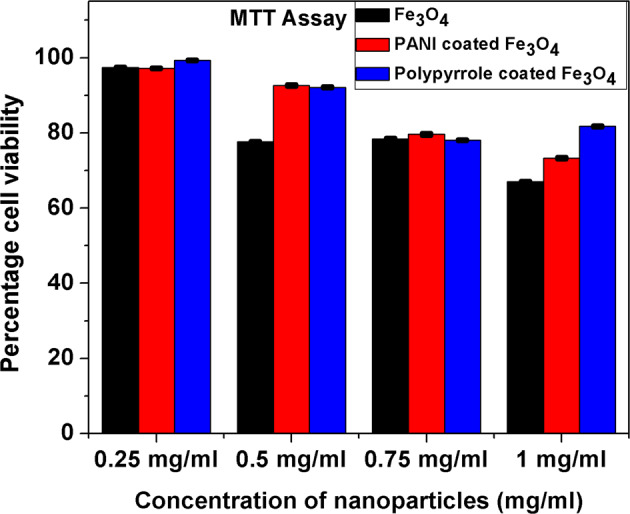
Cytotoxicity studies of MTT assays for different concentrations of Fe_3_O_4_, PANI and polypyrrole-coated Fe_3_O_4_ nanoparticles incubated in U87 cell lines

The viability of U87 cells (Glioma cells) treated with Fe_3_O_4_, PANI- and polypyrrole-coated Fe_3_O_4_ nanoparticles with 0.25, 0.50, 0.75 and 1 mg/ml concentrations is shown in Fig. 8. The cell viability of Fe_3_O_4_ nanoparticles with a concentration of 0.25, 0.50, 0.75 and 1 mg/ml is 97, 77, 78 and 67%, respectively. But in the case of PANI-coated Fe_3_O_4_ nanoparticles, cell viability of 97, 92, 79 and 73% are observed for 0.25, 0.50, 0.75 and 1 mg/ml concentration. But, polypyrrole-coated nanoparticles shows cell viability of 99, 92, 78 and 81%, respectively with 0.25, 0.50, 0.75 and 1 mg/ml concentration of the sample. These observations indicate that all the samples are biocompatible, and the percentage of viable cells decreases with an increase in material concentration. It is interesting to note that 92% of viable cells are seen from PANI- and polypyrrole-coated sample on treating with 0.5 mg/ml of the experimental sample unlike that of Fe_3_O_4_ which shows only 77% of viable cells with same concentration of the sample. These observations reveal that surface functionalization of Fe_3_O_4_ nanoparticles using polyaniline and polypyrrole by r.f. plasma polymerization process favours the enhancement in the biocompatibility of Fe_3_O_4_.

**Fig. 9:**
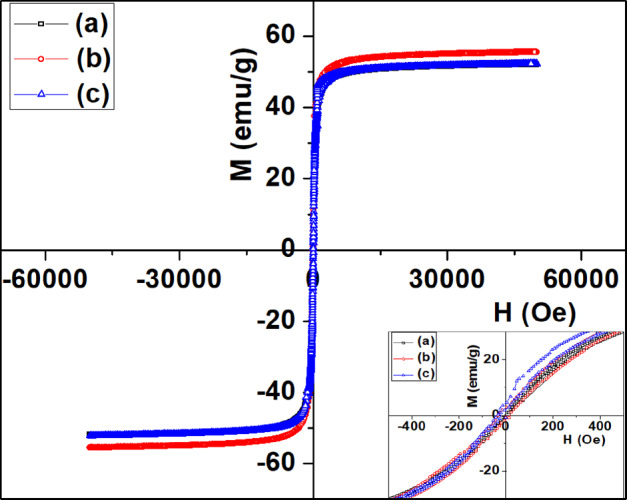
Magnetic hysteresis curves of **a** Fe_3_O_4_, **b** PANI-coated Fe_3_O_4_, and **c** polypyrrole-coated Fe_3_O_4_ at 300 K

Room temperature M–H curve (300 K) of Fe_3_O_4_, PANI-coated and polypyrrole-coated Fe_3_O_4_ nanoparticles shows superparamagnetic behaviour (Fig. 9). The saturation magnetization of Fe_3_O_4_ (M_s_) sample is observed to be 51 emu/gm, which is less than the value reported for bulk magnetite (88 emu/gm). The reduced magnetization of Fe_3_O_4_ may be attributed to the non-contribution of the surface to magnetic property, which arises due to the disordered alignment of surface spins [13, 36]. The saturation magnetization (M_s_) of PANI-coated and polypyrrole-coated Fe_3_O_4_ nanoparticles is 55 and 52 emu/gm respectively. The slight increase in magnetic saturation (M_s_) values of polymer-coated samples is probably attributed to the contact potential induced at the iron-polymer interface [13].

**Fig. 10:**
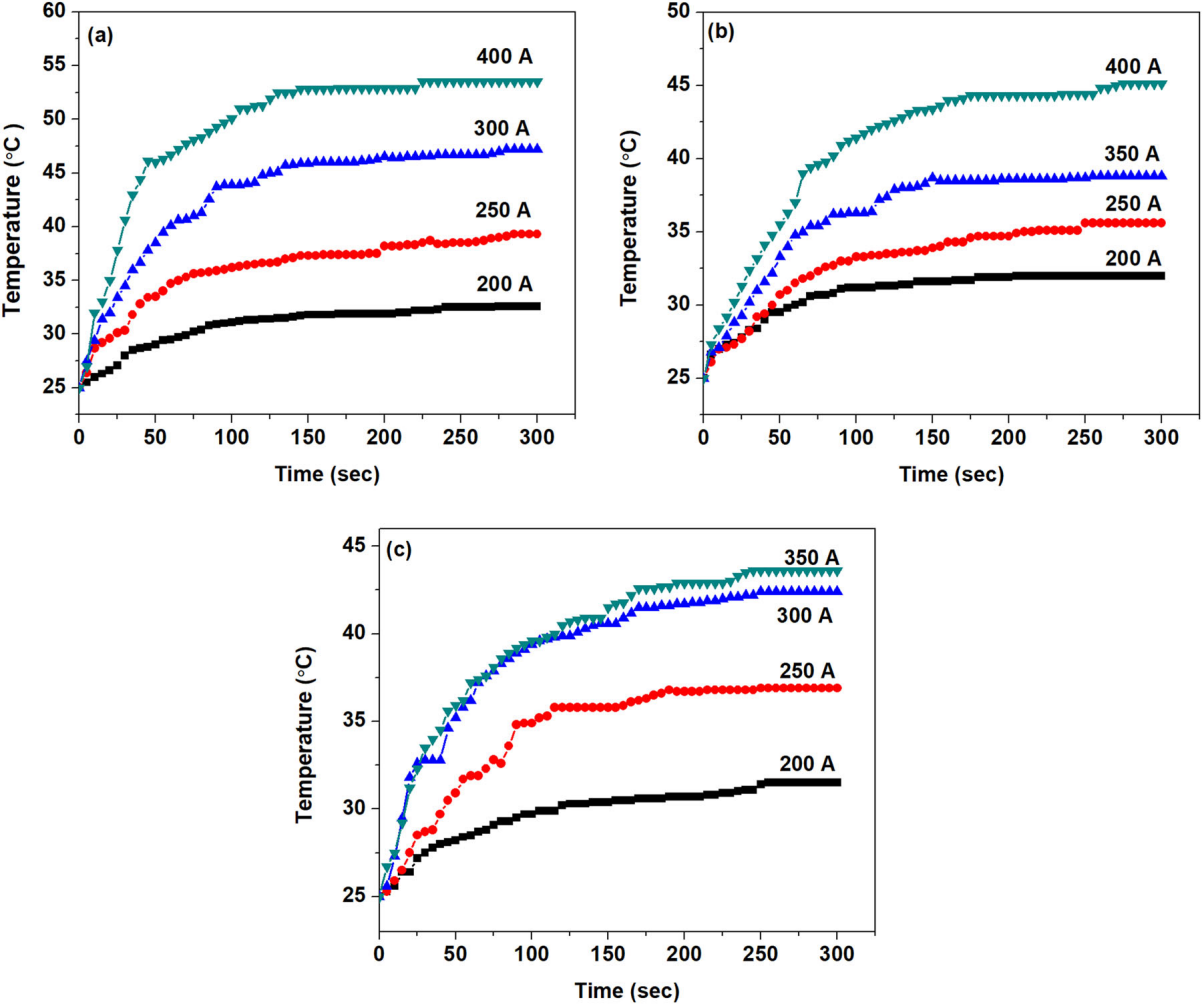
Time-temperature graph of **a** Fe_3_O_4_, **b** PANI-coated Fe_3_O_4_, and **c** polypyrrole-coated Fe_3_O_4_ with 1 mg concentration on exposure to 200–400 A (ac) current with a frequency of 280 kHz

**Fig. 11:**
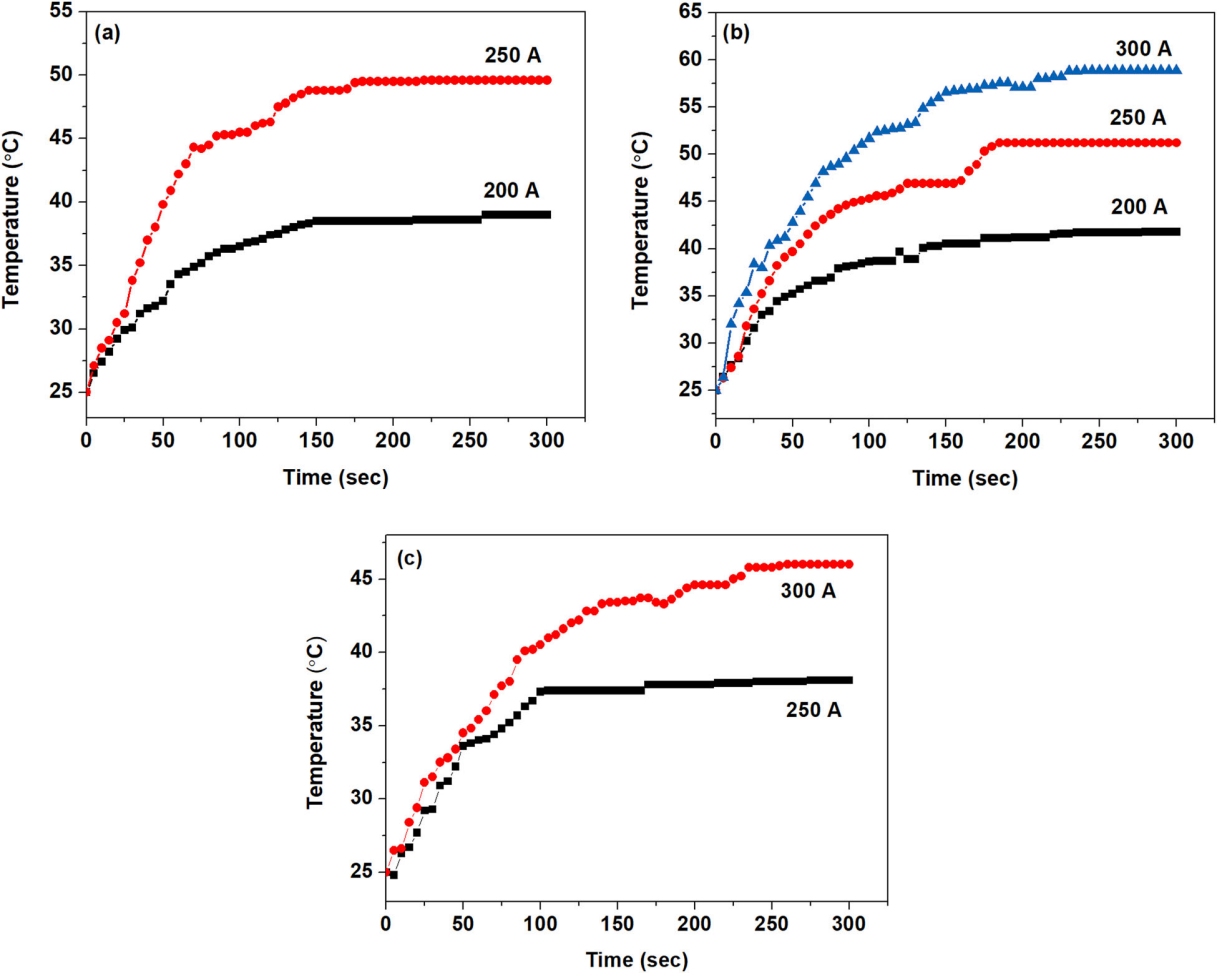
Time–temperature graph of **a** Fe_3_O_4_, **b** PANI-coated Fe_3_O_4_, and **c** polypyrrole-coated Fe_3_O_4_ of 3 mg concentration on exposure to 200–400 A (ac) current with a frequency of 280 kHz

The heating ability of Fe_3_O_4_, PANI and polypyrrole-coated Fe_3_O_4_ nanoparticles with a weight of 1 and 3 mg for about five minutes are evaluated under different current strengths of 200, 250, 300, 350 and 400 A which induces a proportional magnetic field (Figs. 10 and 11). Time–temperature profile of 1 mg Fe_3_O_4_, PANI- and polypyrrole-coated iron oxide magnetic particles (Fig. 10) indicates that the current of 200 A is not enough to generate hyperthermia temperature of 42 °C. Minimum hyperthermia activity is also observed for a current of 250 A for 1 mg concentration of all the 3 samples. But on increasing the alternating magnetic field (AMF) strength *via* adjusting the current strength to 300 A, the 1 mg of Fe_3_O_4_ shows an enhancement in its temperature from 25 to 47 °C. However, polypyrrole-coated iron oxide nanoparticles with 1 mg concentration give an increase in temperature from 25 to 42 °C. Hyperthermia activity is exhibited by PANI-coated iron oxide (1 mg) at a current 400 A and its temperature is raised from 25 to 45 °C.

Hyperthermia activity is absent in all the three samples with 3 mg up to a current of 200 A (Fig. 11). The rise in temperature is observed from 25 to 49 °C for Fe_3_O_4_ and 25 to 51 °C for PANI-coated Fe_3_O_4_ nanoparticles (3 mg) with an applied current of 250 A. However, in the case of 3 mg polypyrrole-coated Fe_3_O_4_, enhancement in temperature is observed from 25 to 46 °C only with a current of 300 A. These studies shows that for achieving hyperthermia activity for a minimum quantity of about 1 mg Fe_3_O_4_ and polypyrrole-coated Fe_3_O_4_ samples, a current of 300 A is required. However, for PANI-coated Fe_3_O_4_, minimum current is slightly higher (400 A) for 1 mg sample with respect to Fe_3_O_4_ and polypyrrole-coated Fe_3_O_4_. Similarly, the current strength of 250 A is minimum to achieve hyperthermia activity from Fe_3_O_4_ and PANI-coated Fe_3_O_4_ with 3 mg sample. But in the case of 3 mg polypyrrole-coated Fe_3_O_4_ slightly higher current strength (300 A) is required to get hyperthermia temperature.

From the above results, one can conclude that Fe_3_O_4_ magnetic nanoparticles show relatively more heating efficiency than polymer-coated magnetite nanoparticles with the same concentration and current strength. The rate of increase in temperature and saturation temperature is related to the applied current strength and the quantity of the experimental sample. However, at a current strength of 250 A (19.33 mT), 3 mg PANI-coated Fe_3_O_4_ samples shows relatively more heating rate (0.293 °C/minute) compared to Fe_3_O_4_ (0.287 °C/minute) and polypyyrole-coated Fe_3_O_4_ (0.168 °C/minute) with same concentration and current. Samples with higher concentration usually show more heating ability and saturation rate as compared to sample with lower concentration with the same current.

Heating efficiency of the samples under investigation is studied by determining specific loss power (SLP) of the particles by suspending a known quantity of samples in distilled water. The sample is subjected to an alternating magnetic field of specific strength and frequency. The change in temperature with respect to time is monitored continuously for a period of three minutes. SLP values of Fe_3_O_4_, PANI and polypyrrole-coated Fe_3_O_4_ nanoparticles is estimated from the following expression [21].

2 $${{{{{\mathrm{SLP}}}}}} = \frac{{CV_{{{\rm{s}}}}}}{{{{{{\mathrm{m}}}}}}}\frac{{dT}}{{dt}}$$

where ‘*C*’ is the volume-specific heat capacity of the sample solution (*C*_water_ = 4185 JL^−1^ K^−1^), ‘*V*_s_’ is the volume of the sample, ‘m’ is the mass of the magnetic nanoparticle present in the sample volume and dT/dt is the initial slope of the temperature versus time curve (Ks^−1^).

Hyperthermia studies based on the applied alternating current, magnetic field strength and SLP of Fe_3_O_4_, PANI-coated Fe_3_O_4_ and Polypyrrole-coated Fe_3_O_4_ at a frequency of 280 kHz is estimated from time-temperature graphs. SLP values are calculated using Eq. 2 and values are shown in Table 1. The values obtained in the present study are comparable with the previous reports [21, 37]. It is observed that for the same current, SLP values of Fe_3_O_4_ nanoparticles are decrease with increase in the concentration of the sample. While for PANI- and polypyrrole-coated Fe_3_O_4_ show an increase in SLP value for higher concentration compared to a low concentration of the sample at constant field strength.

**Table 1:** Specific loss power values of the samples based on hyperthermia studies with an applied alternating current at a frequency of 280 kHz

No.	Current passing through the coil (A)	Magnetic field strength (mT)	Specific loss power of the material concentration (W/g)					
			Fe_3_O_4_		PANI-coated Fe_3_O_4_		Polypyrrole-coated Fe_3_O_4_	
			5 mg/250 μl	10 mg/250 μl	5 mg/250 μl	10 mg/250 μl	5 mg/250 μl	10 mg/250 μl
1.	200	14.4	a	28.98	a	25.95	a	21.51
2.	250	19.33	42.47	35.57	25.32	32.18	20.14	31.39
3.	300	24.17	52.54	49.44	41.53	41.54	20.92	39.86
4.	350	28.99	67.01	b	48.19	b	36.49	b

^a^No temperature rise in that condition

^b^Uncontrolled temperature rise

It is observed that SLP values have direct dependence on magnetic field strength and concentration of the experimental sample (Table 1). SLP values increase with respect to applied field strength for Fe_3_O_4_, PANI and polypyrrole-coated Fe_3_O_4_ with 5 and 10 mg concentration of the sample. Heating efficiency of magnetic nanoparticles has a strong dependence on dipolar–dipolar interactions among the particles. The increase in the quantity of magnetic particles in a system leads to a decrease of interparticle distance, which increases the dipolar–dipolar interactions, results in variations in relaxation time of the nanoparticles [21, 38, 39]. The increase in dipolar-dipolar interactions among the particles enhances the Neel relaxation time consequently SLP values will decrease in agreement with our observation from Fe_3_O_4_ with respect to higher concentration (10 mg)_._

It is observed that polymer capping of Fe_3_O_4_ also plays a major role in the heating efficiency of iron oxide nanoparticles. The hydrodynamic volume of the particle has direct dependence on the thickness of the polymer shell, a thin layer of surfactant coating has significant influence on the relaxation constant of the nanoparticles and also its heat generation capacity [15]. The polymer coating on Fe_3_O_4_ resulted in controlled heat generation unlike that observed in the case of bare Fe_3_O_4_. An increase in SLP value for 10 mg concentration of PANI and polypyrrole-coated Fe_3_O_4_ with respect to 5 mg concentration of the sample with same field strength and frequency is observed. This variation in the heating efficiency of polymer-coated Fe_3_O_4_ NPs is attributed to the relaxation of individual NPs inside the polymer shell which results in an increase in heating rate than uncoated magnetic nanoparticles [23]. Also, the presence of polymer shell can make the matrix rigid and can arise friction between magnetic nanoparticles and shell. This can further generate heat and give higher SLP values for polymer-coated samples at higher concentrations.

Hyperthermia studies show that heat dissipation value of the nanoparticles has a strong dependence on the particle concentration, size, shape anisotropy, strength of the magnetic field and the chemical nature of surface passivating polymers. All these parameters are related to the relaxation process of the superparamagnetic Fe_3_O_4_ nanoparticles which influences the SLP values. TEM images (Figs. 2–5) of the samples used in the investigation also show the presence of spherical as well as rod-shaped particles. Rod-shaped nanoparticles show better hyperthermia performance compared to spherical-shaped ones [40]. Relatively asymmetric morphological spherical and rod-shaped particles can kill cancer cells more effectively than perfect spherical nanoparticles when exposed to an ac magnetic field due to their mechanical oscillations [41]. From this study, it can be concluded that PANI and polypyrrole-coated Fe_3_O_4_ have enhanced hyperthermia efficiency in a temperature-controlled manner compared to bare Fe_3_O_4_ nanoparticles. Present studies reveal that surface passivation of Fe_3_O_4_ with PANI and polypyrrole increases its biocompatibility and thermal stability of Fe_3_O_4_, so this kind of materials can stay back in the blood circulation for a long time. Further, conducting polymers are having a better optical cross-section, which gives PL and fluorescence emission properties. Utilizing the superparamagnetic, optical, fluorescence and hyperthermia properties we can conclude that Fe_3_O_4_, PANI and polypyrrole-coated Fe_3_O_4_ nanoparticles can serve as a good multifunctional material with reduced toxicity and enhanced stability for cancer therapy.

## Conclusions

Surface passivation of superparamagnetic Fe_3_O_4_ nanoparticles can be done with polyaniline and polypyrrole with r.f. polymerization method. PANI and polypyrrole coating over Fe_3_O_4_ nanoparticles increases the thermal stability as well as biocompatibility of the material with fluorescence emission. Time–temperature profile and SLP values of PANI and polypyrrole-coated Fe_3_O_4_ show that they can act as a controlled heat generating system for magnetic hyperthermia applications. Fluorescence emission properties and magnetic hyperthermia activity of the synthesized nanoparticles suggest that they can be used as a promising candidate for cell imaging and the treatment of malignant cells.

## Supplementary information

Supplementary Figure 1

Supplementary Figure 2
